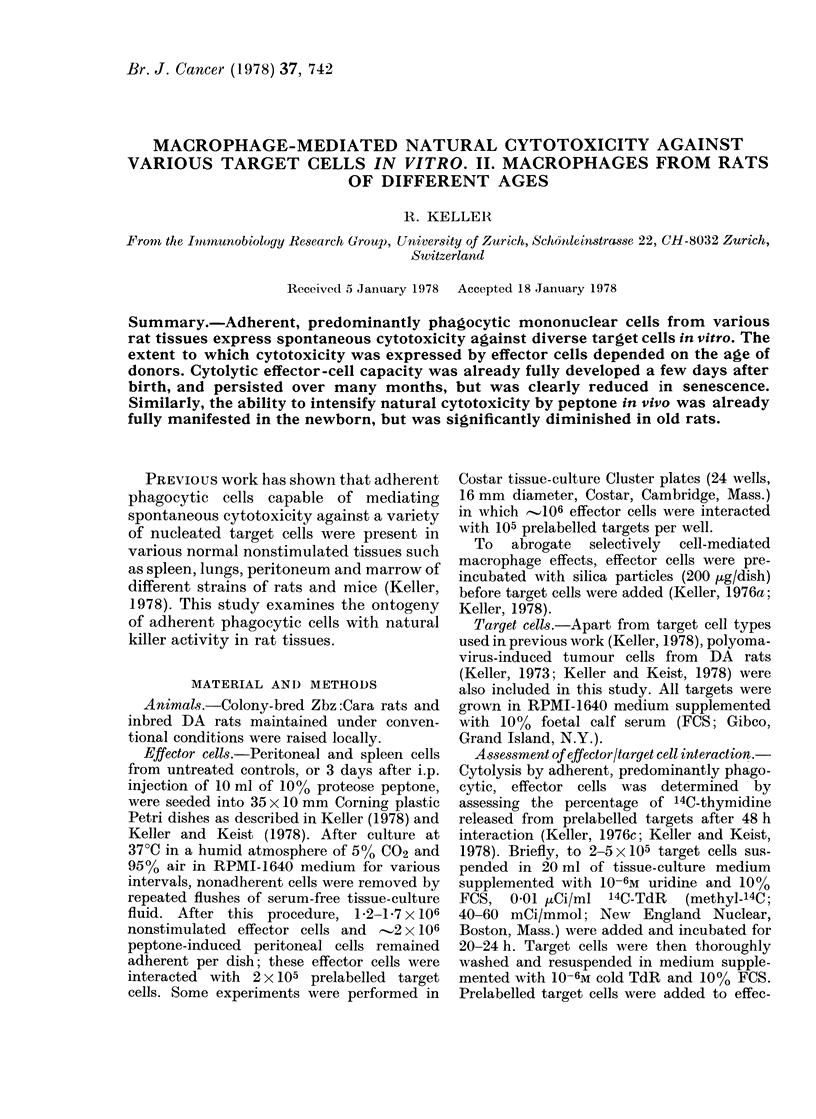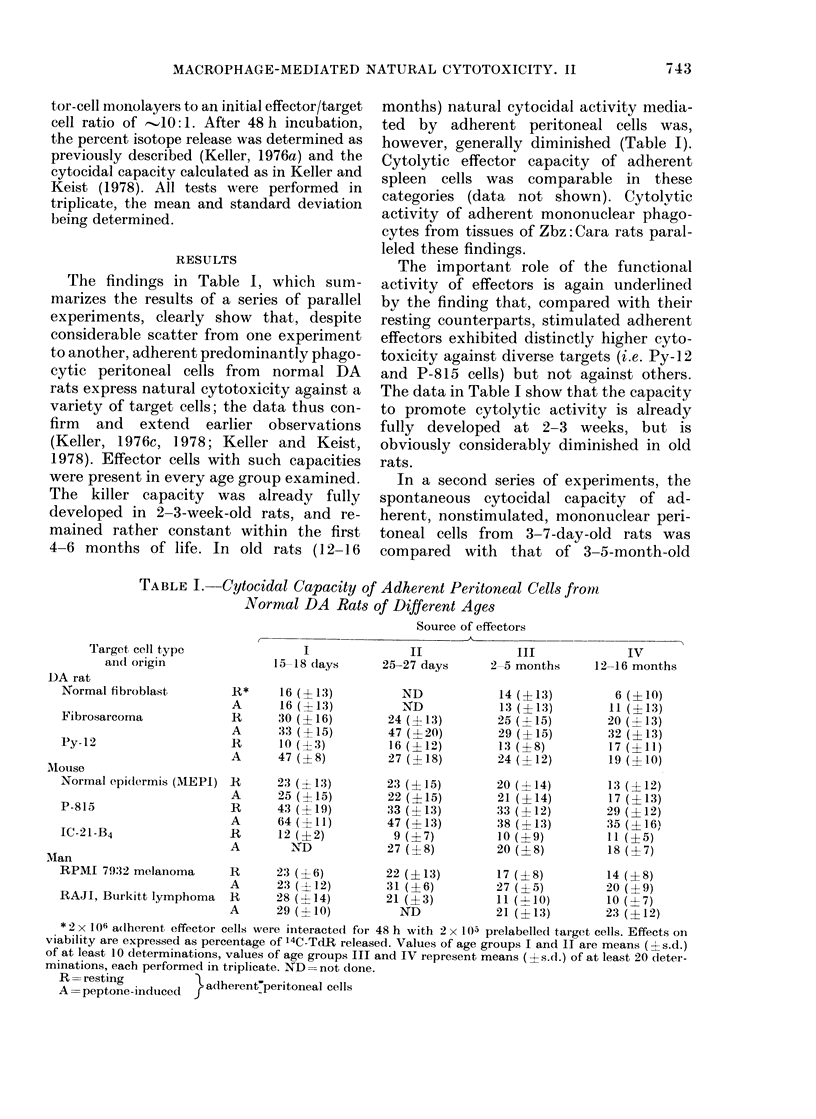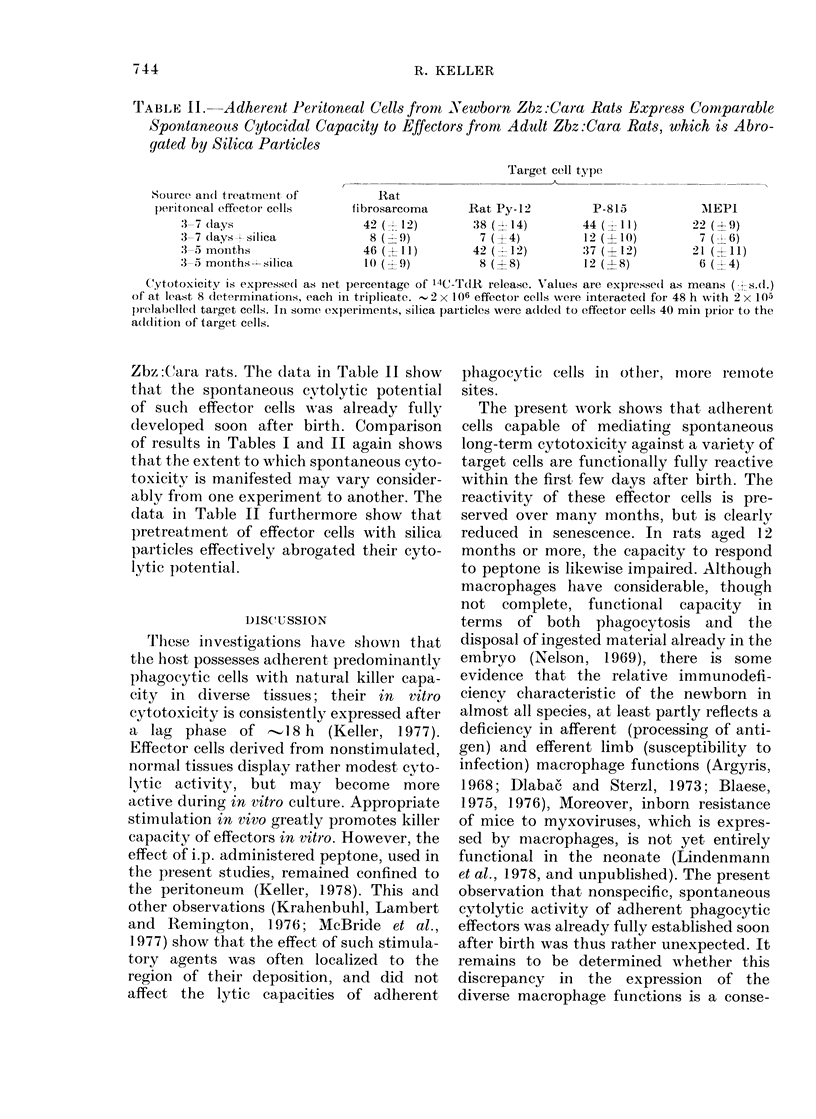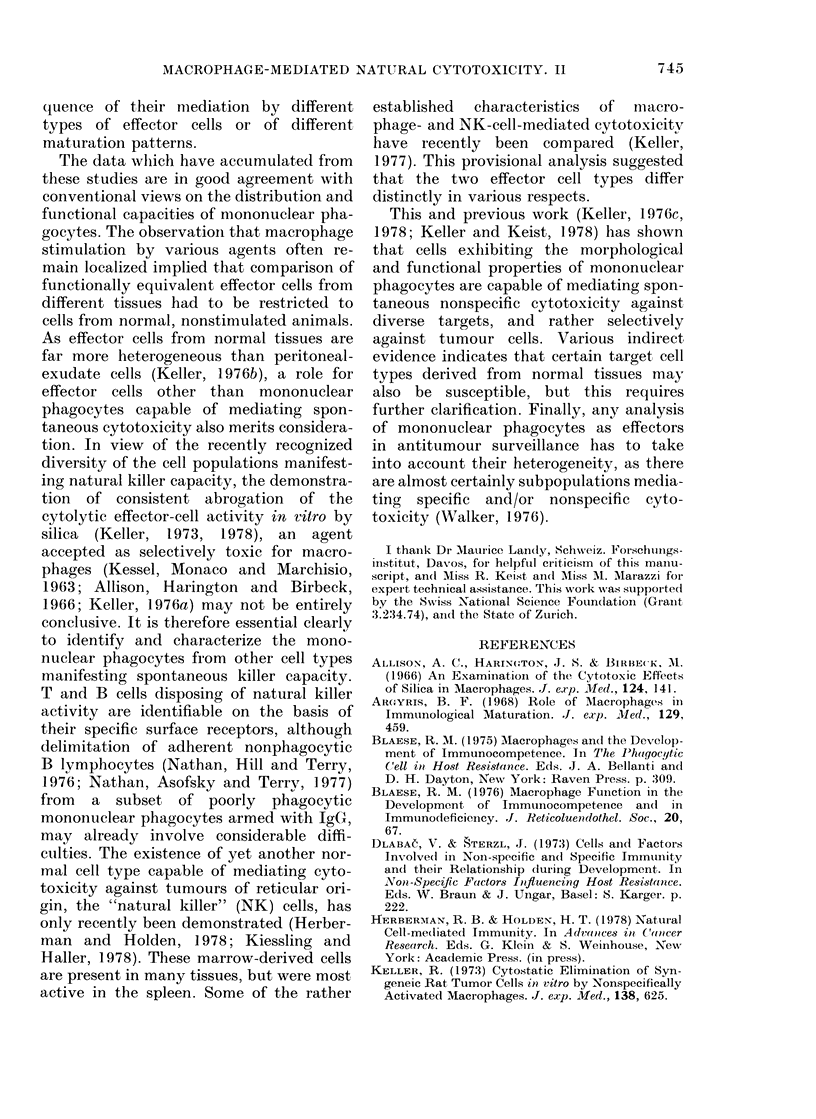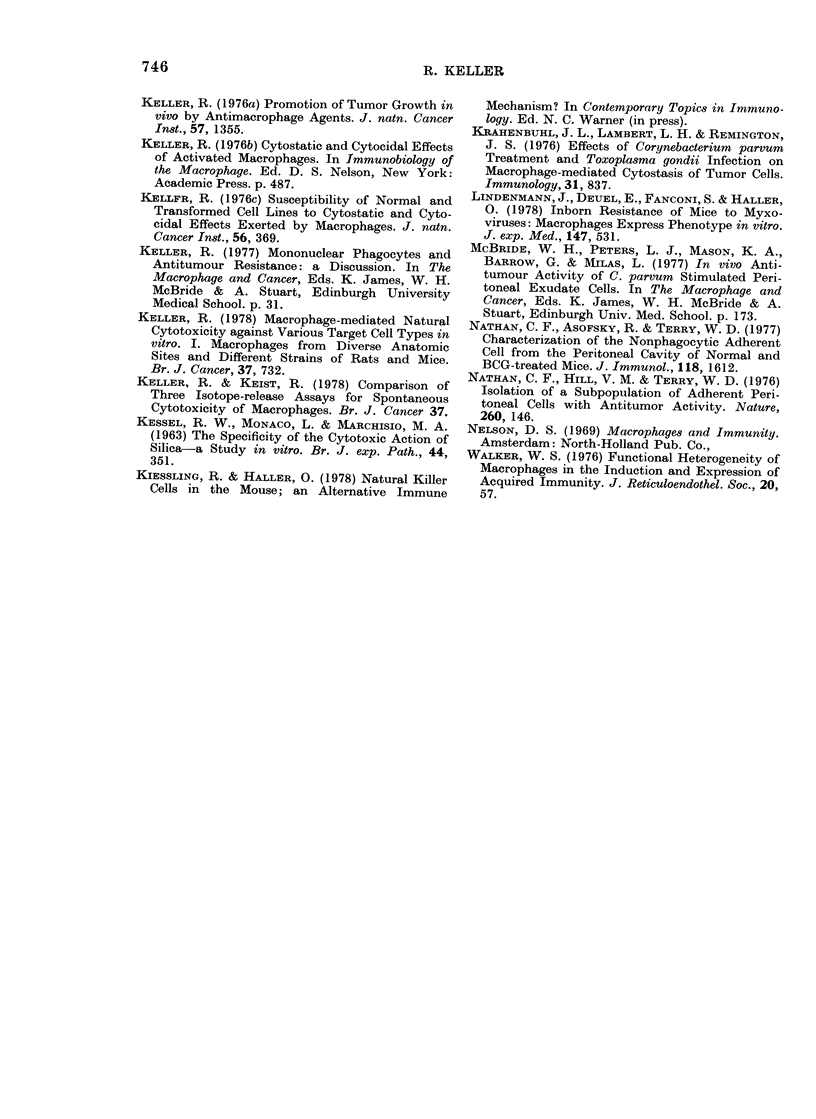# Macrophage-mediated natural cytotoxicity against various target cells in vitro. II. Macrophages from rats of different ages.

**DOI:** 10.1038/bjc.1978.112

**Published:** 1978-05

**Authors:** R. Keller

## Abstract

Adherent, predominantly phagocytic mononuclear cells from various rat tissues express spontaneous cytoxicity against diverse target cells in vitro. The extent to which cytotoxicity was expressed by effector cells depended on the age of donors. Cytolytic effector-cell capacity was already fully developed a few days after birth, and persisted over many months, but was clearly reduced in senescence. Similarly, the ability to intensify natural cytotoxicity by peptone in vivo was already fully manifested in the newborn, but was significantly diminished in old rats.


*Br. J. Cancer* (1978) **37**, 742

### MACROPHAGE-MEDIATED NATURAL CYTOTOXICITY AGAINST VARIOUS TARGET CELLS *IN VITRO*. II. MACROPHAGES FROM RATS OF DIFFERENT AGES

R. KELLER

*From the Immunobiology Research Group, University of Zurich, Schönleinstrasse 22, CH-8032 Zurich, Switzerland*

Received 5 January 1978   Accepted 18 January 1978

**Summary.—Adherent, predominantly phagocytic mononuclear cells from various rat tissues express spontaneous cytotoxicity against diverse target cells *in vitro*. The extent to which cytotoxicity was expressed by effector cells depended on the age of donors. Cytolytic effector-cell capacity was already fully developed a few days after birth, and persisted over many months, but was clearly reduced in senescence. Similarly, the ability to intensify natural cytotoxicity by peptone *in vivo* was already fully manifested in the newborn, but was significantly diminished in old rats.**

PREVIOUS work has shown that adherent phagocytic cells capable of mediating spontaneous cytotoxicity against a variety of nucleated target cells were present in various normal nonstimulated tissues such as spleen, lungs, peritoneum and marrow of different strains of rats and mice (Keller, 1978). This study examines the ontogeny of adherent phagocytic cells with natural killer activity in rat tissues.

### MATERIAL AND METHODS

*Animals.*—Colony-bred Zbz:Cara rats and inbred DA rats maintained under conventional conditions were raised locally.

*Effector cells.*—Peritoneal and spleen cells from untreated controls, or 3 days after i.p. injection of 10 ml of 10% proteose peptone, were seeded into $35 \times 10$ mm Corning plastic Petri dishes as described in Keller (1978) and Keller and Keist (1978). After culture at 37°C in a humid atmosphere of 5% $CO_2$ and 95% air in RPMI-1640 medium for various intervals, nonadherent cells were removed by repeated flushes of serum-free tissue-culture fluid. After this procedure, $1{\cdot}2\text{–}1{\cdot}7 \times 10^6$ nonstimulated effector cells and $\sim 2 \times 10^6$ peptone-induced peritoneal cells remained adherent per dish; these effector cells were interacted with $2 \times 10^5$ prelabelled target cells. Some experiments were performed in Costar tissue-culture Cluster plates (24 wells, 16 mm diameter, Costar, Cambridge, Mass.) in which $\sim 10^6$ effector cells were interacted with $10^5$ prelabelled targets per well.

To abrogate selectively cell-mediated macrophage effects, effector cells were preincubated with silica particles (200 $\mu$g/dish) before target cells were added (Keller, 1976*a*; Keller, 1978).

*Target cells.*—Apart from target cell types used in previous work (Keller, 1978), polyomavirus-induced tumour cells from DA rats (Keller, 1973; Keller and Keist, 1978) were also included in this study. All targets were grown in RPMI-1640 medium supplemented with 10% foetal calf serum (FCS; Gibco, Grand Island, N.Y.).

*Assessment of effector/target cell interaction.*—Cytolysis by adherent, predominantly phagocytic, effector cells was determined by assessing the percentage of $^{14}C$-thymidine released from prelabelled targets after 48 h interaction (Keller, 1976*c*; Keller and Keist, 1978). Briefly, to $2\text{–}5 \times 10^5$ target cells suspended in 20 ml of tissue-culture medium supplemented with $10^{-6}$M uridine and 10% FCS, 0·01 $\mu$Ci/ml $^{14}C$-TdR (methyl-$^{14}C$; 40–60 mCi/mmol; New England Nuclear, Boston, Mass.) were added and incubated for 20–24 h. Target cells were then thoroughly washed and resuspended in medium supplemented with $10^{-6}$M cold TdR and 10% FCS. Prelabelled target cells were added to effector-cell monolayers to an initial effector/target cell ratio of ~10:1. After 48 h incubation, the percent isotope release was determined as previously described (Keller, 1976*a*) and the cytocidal capacity calculated as in Keller and Keist (1978). All tests were performed in triplicate, the mean and standard deviation being determined.

### RESULTS

The findings in Table I, which summarizes the results of a series of parallel experiments, clearly show that, despite considerable scatter from one experiment to another, adherent predominantly phagocytic peritoneal cells from normal DA rats express natural cytotoxicity against a variety of target cells; the data thus confirm and extend earlier observations (Keller, 1976*c*, 1978; Keller and Keist, 1978). Effector cells with such capacities were present in every age group examined. The killer capacity was already fully developed in 2–3-week-old rats, and remained rather constant within the first 4–6 months of life. In old rats (12–16 months) natural cytocidal activity mediated by adherent peritoneal cells was, however, generally diminished (Table I). Cytolytic effector capacity of adherent spleen cells was comparable in these categories (data not shown). Cytolytic activity of adherent mononuclear phagocytes from tissues of Zbz:Cara rats paralleled these findings.

The important role of the functional activity of effectors is again underlined by the finding that, compared with their resting counterparts, stimulated adherent effectors exhibited distinctly higher cytotoxicity against diverse targets (*i.e.* Py-12 and P-815 cells) but not against others. The data in Table I show that the capacity to promote cytolytic activity is already fully developed at 2–3 weeks, but is obviously considerably diminished in old rats.

In a second series of experiments, the spontaneous cytocidal capacity of adherent, nonstimulated, mononuclear peritoneal cells from 3–7-day-old rats was compared with that of 3–5-month-old

TABLE I.—*Cytocidal Capacity of Adherent Peritoneal Cells from Normal DA Rats of Different Ages*

| Target cell type and origin | | Source of effectors: I 15–18 days | II 25–27 days | III 2–5 months | IV 12–16 months |
|---|---|---|---|---|---|
| DA rat | | | | | |
| Normal fibroblast | R* | 16 (±13) | ND | 14 (±13) | 6 (±10) |
| | A | 16 (±13) | ND | 13 (±13) | 11 (±13) |
| Fibrosarcoma | R | 30 (±16) | 24 (±13) | 25 (±15) | 20 (±13) |
| | A | 33 (±15) | 47 (±20) | 29 (±15) | 32 (±13) |
| Py-12 | R | 10 (±3) | 16 (±12) | 13 (±8) | 17 (±11) |
| | A | 47 (±8) | 27 (±18) | 24 (±12) | 19 (±10) |
| Mouse | | | | | |
| Normal epidermis (MEPI) | R | 23 (±13) | 23 (±15) | 20 (±14) | 13 (±12) |
| | A | 25 (±15) | 22 (±15) | 21 (±14) | 17 (±13) |
| P-815 | R | 43 (±19) | 33 (±13) | 33 (±12) | 29 (±12) |
| | A | 64 (±11) | 47 (±13) | 38 (±13) | 35 (±16) |
| IC-21-$B_4$ | R | 12 (±2) | 9 (±7) | 10 (±9) | 11 (±5) |
| | A | ND | 27 (±8) | 20 (±8) | 18 (±7) |
| Man | | | | | |
| RPMI 7932 melanoma | R | 23 (±6) | 22 (±13) | 17 (±8) | 14 (±8) |
| | A | 23 (±12) | 31 (±6) | 27 (±5) | 20 (±9) |
| RAJI, Burkitt lymphoma | R | 28 (±14) | 21 (±3) | 11 (±10) | 10 (±7) |
| | A | 29 (±10) | ND | 21 (±13) | 23 (±12) |

*$2 \times 10^6$ adherent effector cells were interacted for 48 h with $2 \times 10^5$ prelabelled target cells. Effects on viability are expressed as percentage of $^{14}$C-TdR released. Values of age groups I and II are means (±s.d.) of at least 10 determinations, values of age groups III and IV represent means (±s.d.) of at least 20 determinations, each performed in triplicate. ND = not done.

R = resting } adherent peritoneal cells

A = peptone-induced }

TABLE II.—*Adherent Peritoneal Cells from Newborn Zbz:Cara Rats Express Comparable Spontaneous Cytocidal Capacity to Effectors from Adult Zbz:Cara Rats, which is Abrogated by Silica Particles*

| Source and treatment of peritoneal effector cells | Target cell type: Rat fibrosarcoma | Rat Py-12 | P-815 | MEPI |
|---|---|---|---|---|
| 3–7 days | 42 (±12) | 38 (±14) | 44 (±11) | 22 (±9) |
| 3–7 days+silica | 8 (±9) | 7 (±4) | 12 (±10) | 7 (±6) |
| 3–5 months | 46 (±11) | 42 (±12) | 37 (±12) | 21 (±11) |
| 3–5 months+silica | 10 (±9) | 8 (±8) | 12 (±8) | 6 (±4) |

Cytotoxicity is expressed as net percentage of $^{14}$C-TdR release. Values are expressed as means (±s.d.) of at least 8 determinations, each in triplicate. $\sim 2 \times 10^6$ effector cells were interacted for 48 h with $2 \times 10^5$ prelabelled target cells. In some experiments, silica particles were added to effector cells 40 min prior to the addition of target cells.

Zbz:Cara rats. The data in Table II show that the spontaneous cytolytic potential of such effector cells was already fully developed soon after birth. Comparison of results in Tables I and II again shows that the extent to which spontaneous cytotoxicity is manifested may vary considerably from one experiment to another. The data in Table II furthermore show that pretreatment of effector cells with silica particles effectively abrogated their cytolytic potential.

### DISCUSSION

These investigations have shown that the host possesses adherent predominantly phagocytic cells with natural killer capacity in diverse tissues; their *in vitro* cytotoxicity is consistently expressed after a lag phase of ~18 h (Keller, 1977). Effector cells derived from nonstimulated, normal tissues display rather modest cytolytic activity, but may become more active during *in vitro* culture. Appropriate stimulation *in vivo* greatly promotes killer capacity of effectors *in vitro*. However, the effect of i.p. administered peptone, used in the present studies, remained confined to the peritoneum (Keller, 1978). This and other observations (Krahenbuhl, Lambert and Remington, 1976; McBride *et al.*, 1977) show that the effect of such stimulatory agents was often localized to the region of their deposition, and did not affect the lytic capacities of adherent phagocytic cells in other, more remote sites.

The present work shows that adherent cells capable of mediating spontaneous long-term cytotoxicity against a variety of target cells are functionally fully reactive within the first few days after birth. The reactivity of these effector cells is preserved over many months, but is clearly reduced in senescence. In rats aged 12 months or more, the capacity to respond to peptone is likewise impaired. Although macrophages have considerable, though not complete, functional capacity in terms of both phagocytosis and the disposal of ingested material already in the embryo (Nelson, 1969), there is some evidence that the relative immunodeficiency characteristic of the newborn in almost all species, at least partly reflects a deficiency in afferent (processing of antigen) and efferent limb (susceptibility to infection) macrophage functions (Argyris, 1968; Dlabač and Sterzl, 1973; Blaese, 1975, 1976), Moreover, inborn resistance of mice to myxoviruses, which is expressed by macrophages, is not yet entirely functional in the neonate (Lindenmann *et al.*, 1978, and unpublished). The present observation that nonspecific, spontaneous cytolytic activity of adherent phagocytic effectors was already fully established soon after birth was thus rather unexpected. It remains to be determined whether this discrepancy in the expression of the diverse macrophage functions is a consequence of their mediation by different types of effector cells or of different maturation patterns.

The data which have accumulated from these studies are in good agreement with conventional views on the distribution and functional capacities of mononuclear phagocytes. The observation that macrophage stimulation by various agents often remain localized implied that comparison of functionally equivalent effector cells from different tissues had to be restricted to cells from normal, nonstimulated animals. As effector cells from normal tissues are far more heterogeneous than peritoneal-exudate cells (Keller, 1976*b*), a role for effector cells other than mononuclear phagocytes capable of mediating spontaneous cytotoxicity also merits consideration. In view of the recently recognized diversity of the cell populations manifesting natural killer capacity, the demonstration of consistent abrogation of the cytolytic effector-cell activity *in vitro* by silica (Keller, 1973, 1978), an agent accepted as selectively toxic for macrophages (Kessel, Monaco and Marchisio, 1963; Allison, Harington and Birbeck, 1966; Keller, 1976*a*) may not be entirely conclusive. It is therefore essential clearly to identify and characterize the mononuclear phagocytes from other cell types manifesting spontaneous killer capacity. T and B cells disposing of natural killer activity are identifiable on the basis of their specific surface receptors, although delimitation of adherent nonphagocytic B lymphocytes (Nathan, Hill and Terry, 1976; Nathan, Asofsky and Terry, 1977) from a subset of poorly phagocytic mononuclear phagocytes armed with IgG, may already involve considerable difficulties. The existence of yet another normal cell type capable of mediating cytotoxicity against tumours of reticular origin, the "natural killer" (NK) cells, has only recently been demonstrated (Herberman and Holden, 1978; Kiessling and Haller, 1978). These marrow-derived cells are present in many tissues, but were most active in the spleen. Some of the rather established characteristics of macrophage- and NK-cell-mediated cytotoxicity have recently been compared (Keller, 1977). This provisional analysis suggested that the two effector cell types differ distinctly in various respects.

This and previous work (Keller, 1976*c*, 1978; Keller and Keist, 1978) has shown that cells exhibiting the morphological and functional properties of mononuclear phagocytes are capable of mediating spontaneous nonspecific cytotoxicity against diverse targets, and rather selectively against tumour cells. Various indirect evidence indicates that certain target cell types derived from normal tissues may also be susceptible, but this requires further clarification. Finally, any analysis of mononuclear phagocytes as effectors in antitumour surveillance has to take into account their heterogeneity, as there are almost certainly subpopulations mediating specific and/or nonspecific cytotoxicity (Walker, 1976).

I thank Dr Maurice Landy, Schweiz. Forschungsinstitut, Davos, for helpful criticism of this manuscript, and Miss R. Keist and Miss M. Marazzi for expert technical assistance. This work was supported by the Swiss National Science Foundation (Grant 3.234.74), and the State of Zurich.

### REFERENCES

Allison, A. C., Harington, J. S. & Birbeck, M. (1966) An Examination of the Cytotoxic Effects of Silica in Macrophages. *J. exp. Med.*, **124**, 141.

Argyris, B. F. (1968) Role of Macrophages in Immunological Maturation. *J. exp. Med.*, **129**, 459.

Blaese, R. M. (1975) Macrophages and the Development of Immunocompetence. In *The Phagocytic Cell in Host Resistance*. Eds. J. A. Bellanti and D. H. Dayton, New York: Raven Press. p. 309.

Blaese, R. M. (1976) Macrophage Function in the Development of Immunocompetence and in Immunodeficiency. *J. Reticoluendothel. Soc.*, **20**, 67.

Dlabač, V. & Šterzl, J. (1973) Cells and Factors Involved in Non-specific and Specific Immunity and their Relationship during Development. In *Non-Specific Factors Influencing Host Resistance*. Eds. W. Braun & J. Ungar, Basel: S. Karger. p. 222.

Herberman, R. B. & Holden, H. T. (1978) Natural Cell-mediated Immunity. In *Advances in Cancer Research*. Eds. G. Klein & S. Weinhouse, New York: Academic Press. (in press).

Keller, R. (1973) Cytostatic Elimination of Syngeneic Rat Tumor Cells *in vitro* by Nonspecifically Activated Macrophages. *J. exp. Med.*, **138**, 625.

KELLER, R. (1976*a*) Promotion of Tumor Growth *in vivo* by Antimacrophage Agents. *J. natn. Cancer Inst.*, **57**, 1355.

KELLER, R. (1976*b*) Cytostatic and Cytocidal Effects of Activated Macrophages. In *Immunobiology of the Macrophage*. Ed. D. S. Nelson, New York: Academic Press. p. 487.

KELLFR, R. (1976*c*) Susceptibility of Normal and Transformed Cell Lines to Cytostatic and Cytocidal Effects Exerted by Macrophages. *J. natn. Cancer Inst.*, **56**, 369.

KELLER, R. (1977) Mononuclear Phagocytes and Antitumour Resistance: a Discussion. In *The Macrophage and Cancer*, Eds. K. James, W. H. McBride & A. Stuart, Edinburgh University Medical School. p. 31.

KELLER, R. (1978) Macrophage-mediated Natural Cytotoxicity against Various Target Cell Types *in vitro*. I. Macrophages from Diverse Anatomic Sites and Different Strains of Rats and Mice. *Br. J. Cancer*, **37**, 732.

KELLER, R. & KEIST, R. (1978) Comparison of Three Isotope-release Assays for Spontaneous Cytotoxicity of Macrophages. *Br. J. Cancer* **37.**

KESSEL, R. W., MONACO, L. & MARCHISIO, M. A. (1963) The Specificity of the Cytotoxic Action of Silica—a Study *in vitro*. *Br. J. exp. Path.*, **44**, 351.

KIESSLING, R. & HALLER, O. (1978) Natural Killer Cells in the Mouse; an Alternative Immune Mechanism? In *Contemporary Topics in Immunology*. Ed. N. C. Warner (in press).

KRAHENBUHL, J. L., LAMBERT, L. H. & REMINGTON, J. S. (1976) Effects of *Corynebacterium parvum* Treatment and *Toxoplasma gondii* Infection on Macrophage-mediated Cytostasis of Tumor Cells. *Immunology*, **31**, 837.

LINDENMANN, J., DEUEL, E., FANCONI, S. & HALLER, O. (1978) Inborn Resistance of Mice to Myxoviruses: Macrophages Express Phenotype *in vitro*. *J. exp. Med.*, **147**, 531.

MCBRIDE, W. H., PETERS, L. J., MASON, K. A., BARROW, G. & MILAS, L. (1977) *In vivo* Antitumour Activity of *C. parvum* Stimulated Peritoneal Exudate Cells. In *The Macrophage and Cancer*, Eds. K. James, W. H. McBride & A. Stuart, Edinburgh Univ. Med. School. p. 173.

NATHAN, C. F., ASOFSKY, R. & TERRY, W. D. (1977) Characterization of the Nonphagocytic Adherent Cell from the Peritoneal Cavity of Normal and BCG-treated Mice. *J. Immunol.*, **118**, 1612.

NATHAN, C. F., HILL, V. M. & TERRY, W. D. (1976) Isolation of a Subpopulation of Adherent Peritoneal Cells with Antitumor Activity. *Nature*, **260**, 146.

NELSON, D. S. (1969) *Macrophages and Immunity*. Amsterdam: North-Holland Pub. Co.,

WALKER, W. S. (1976) Functional Heterogeneity of Macrophages in the Induction and Expression of Acquired Immunity. *J. Reticuloendothel. Soc.*, **20**, 57.